# Past and projected growth of Australia’s older migrant populations

**DOI:** 10.1186/s41118-020-00091-6

**Published:** 2020-08-05

**Authors:** Tom Wilson, Peter McDonald, Jeromey Temple, Bianca Brijnath, Ariane Utomo

**Affiliations:** 1 Demography and Ageing Unit, Melbourne School of Population and Global Health, University of Melbourne, 207 Bouverie St, Melbourne, Victoria 3010 Australia; 2 National Ageing Research Institute (NARI), Parkville, Australia; 3 School of Geography, University of Melbourne, Melbourne, Australia

**Keywords:** Australia, Immigrant populations, Country of birth, Population projections, Older population, Population ageing, Diversity

## Abstract

In recent years, Australia’s older population (aged 65 and over) has been growing rapidly, accompanied by a shift in its country of birth composition. Although a great deal of research has been undertaken on past and current aspects of Australia’s migrant groups, little attention has been paid to future demographic trends in older populations. The aim of this paper is to examine recent and possible future demographic trends of Australia’s migrant populations at the older ages. We present population estimates by country and broad global region of birth from 1996 to 2016, and then new birthplace-specific population projections for the 2016 to 2056 period. Our findings show that substantial growth of the 65+ population will occur in the coming decades, and that the overseas-born will shift from a Europe-born dominance to an Asia-born dominance. Cohort flow (the effect of varying sizes of cohorts moving into the 65+ age group over time) will be the main driver of growth for most older birthplace populations. The shifting demography of Australia’s older population signals many policy, planning, service delivery and funding challenges for government and private sector providers. We discuss those related to aged care, health care, language services, the aged care workforce, regulatory frameworks and future research needs in demography and gerontology.

## Introduction

Like most high-income countries, Australia’s population is experiencing population ageing—both numerical ageing in which the size of the population in older age groups is growing, and structural ageing in which the share of the total population in those age groups is rising. We choose the conventional definition of the older population as those aged 65 years and above, but acknowledge that alternative definitions are growing in popularity (Sanderson & Scherbov, 2019). The composition of the older population, in terms of country of birth, language, religion, living arrangements, health, wealth, education and other socio-economic characteristics, is also evolving.

In this paper, we focus on the recent past and projected future of Australia’s older population by country (or global region) of birth. Country of birth is important because of its association with English language proficiency, which is central to social participation and ability to access government services in Australia (McDonald, Moyle, & Temple, 2019). A sizeable body of literature had found that older migrants from culturally and linguistically diverse backgrounds encounter multiple barriers in accessing aged-care and health-related services in Australia (Haralambous et al., 2014; Radermacher, Feldman, & Browning, 2009; Rao, Warburton, & Bartlett, 2006; Tipping & Whiteside, 2015). Indeed, for many non-English speaking countries of birth, substantial proportions of the population aged 65+ were recorded in the 2016 Census as speaking English either “not well” or “not at all”. Table 1 shows the spoken English language proficiency in Australia by broad global region of birth.

**Table 1:** Percentage of people aged 65+ by spoken English language proficiency, 2016

Broad region of birth	Very well or well	Not well or not at all
Oceania	89	11
North-West Europe	96	4
Southern and Eastern Europe	67	33
North Africa and the Middle East	61	39
South-East Asia	56	44
North-East Asia	31	69
Southern and Central Asia	68	32
Americas	68	32
Sub-Saharan Africa	89	11

Source: ABS 2016 Census

Note: See Appendix Table 4 for the definition of broad regions

Surprisingly for a country with such a long and rich immigration history, little attention has been given to the future of Australia’s older migrant populations. This stands in contrast to a voluminous literature on historical and contemporary aspects of Australia’s immigrant populations (Borrie, 1994; Hugo, 2004, 2006; Jupp, 2001, 2002; Khoo, 2003; Khoo & McDonald, 2003; Price, 1987, 1996; Raymer, Shi, Guan, Baffour, & Wilson, 2018). With respect to projections, the Australian Bureau of Statistics (ABS) produces projections of Australia’s population as a whole and its Aboriginal and Torres Strait Islander population every few years (Australian Bureau of Statistics, 2018, 2019a) but not by birthplace or ethnicity. The last projections with birthplace or ethnicity details were published some time ago, before the sharp increase in immigration in the early twenty-first century. Gibson, Braun, Benham, and Mason (2001) prepared projections of Australia’s older population from culturally and linguistically diverse (CALD) backgrounds for the period 1996 to 2026 assuming no international migration. Prior to that Price (1996) created projections by birthplace and “ethnic strength” for the period 1991 to 2025. Projections by country of birth or ethnic group are more common in Canada, the USA, New Zealand and the UK. The national statistical offices of Canada, New Zealand, and the USA include such characteristics as part of their regular national population projections (for example Dion, Caron-Malenfant, Grondin, & Grenier, 2015; Statistics New Zealand, 2017; United States Census Bureau., 2018). For the UK, academic researchers have created several sets of ethnic group population projections in recent years (Lomax, Wohland, Rees, & Norman, 2020; Rees, Wohland, Norman, Lomax, & Clark, 2017).

In the context of ageing and migration in Australia, projections of the future overseas-born population serve to inform research, policy, and practice around health promotion (Clarke & Isphording, 2017; Henderson & Kendall, 2011), culturally appropriate aged-care services (Radermacher et al., 2009), and general health care and social exclusion policies (Rao et al., 2006; Warburton, Bartlett, & Rao, 2009). More broadly, projections may also contribute to the growing international scholarship on migration-driven change in employment patterns, demands for social security and support services, and overall welfare and living standards in migrant-receiving high income countries (Bolzman, Poncioni-Derigo, Vial, & Fibbi, 2004; De Alwis, Parr, & Guo, 2019; Mui, Nguyen, Kang, & Domanski, 2006; Parr & Guest, 2019; Rallu, 2017; Warnes, Friedrich, Kellaher, & Torres, 2004). Our paper contributes to this literature by presenting new projections of Australia’s older population by birthplace from 2016 to 2056. It seeks to address the following research questions:

1. How has Australia’s population aged 65+ by birthplace changed in recent years?
2. How might it change in the future?
3. What are the demographic drivers of these projected changes?

Following this introduction, we provide some historical context to the study by outlining the main features of Australia’s post-war immigration policies and trends. Then, we describe the input data, assumptions and projection methods employed. Next, we describe the recent past and projected future of Australia’s older populations by birthplace and the demographic drivers of future growth. The implications of these coming demographic changes for policy, service provision and research form the focus on the “Discussion” section, before we finish with a final section of concluding remarks.

## Historical context

The birthplace composition of Australia’s contemporary older population reflects the waves of migration from different countries since the end of the Second World War. In 1947, only two percent of the Australian population had origins outside of Australia, New Zealand and the British Isles (McDonald, 2019). Thus, the very oldest people today who were born overseas come from the British Isles in particular, but also from New Zealand. Migration from Britain and New Zealand has continued at a relatively high level throughout the past 70 years meaning that today, migrants for these origins are spread across the age range. This means that they continue to contribute to the aged population throughout the projection period.

Immediately after the Second World War, as part of Australia’s new and vigorous post-war migration programme, migrants began arriving in very large numbers from Western and Eastern Europe (Markus, Jupp, & McDonald, 2009). Many were displaced persons representing a ready source of new immigrants. For many countries in these regions, there was very little migration after 1960. Thus, migrants born in countries such as Germany, the Netherlands and Hungary are now heavily concentrated in the oldest ages.

The next main wave of non-British immigrants came from Southern Europe especially from Italy, Malta and Greece in the 1950s and 1960s (McDonald, 2000a, 2000b, 2000c). With little further migration from these countries from 1970 onwards, these birthplace groups are also very old today. Unlike the earlier migrants from Western and Eastern Europe, very few of these Southern European migrants were educated and most worked in manufacturing and construction jobs for which proficiency in English was not a major requirement. Consequently, many today have very limited English proficiency (McDonald et al., 2019) and this has major implications for the provision to them of social and health services. This is the group for whom ethnic-specific service provision is most important in Australia at present. On the other hand, older Southern Europeans are more likely to own their own houses than any other birthplace group including the Australian-born (McDonald & Moyle, 2019).

Migration from the former Yugoslavia was significant in the early post-war refugee flows but it commenced again in the late 1960s and 1970s and then again after the civil unrest related to the breakup of Yugoslavia (Kosovich, 2001). Thus, migrants from the former Yugoslavian countries were spread across a relatively wide range of ages in 2016. This is also true to some extent of those born in Poland who arrived in two waves, the early post-war years and in the 1980s.

When the White Australia Policy was abolished in the early 1970s, from the late 1970s into the 1980s, a very large number of refugees from Vietnam were resettled in Australia (Coughlan, 2001). Most were young, but some were relatively old upon arrival. In the twenty-first century, immigrants to Australia have come primarily from Asian countries, particularly India and China. Often arriving as students, they then converted to permanent residence onshore. Most were still relatively young in 2016 but many have assisted older relatives migrate to Australia thus contributing to the large numbers at older ages. English-language proficiency is a major issue for older persons from China who have also arrived relatively late in their lives (McDonald et al., 2019). This group will pose challenges for service provision for older people in the coming years.

## Data and methods

Estimated resident populations (ERPs) by country of birth by sex and 5-year age group were obtained from the Australian Bureau of Statistics (ABS) for the years 1996 to 2016 (Australian Bureau of Statistics, 2019b). ERPs are considered the best estimate of the ‘true’ resident population; they are generally slightly higher than census counts because they have been adjusted for residents missed by the census. For the population projections, which launch from 2016, more detailed 2016 jump-off populations were required than those available from the ABS. Five-year age group ERPs were disaggregated to single years of age using detailed 2016 Census data constrained to published ERP statistics by means of iterative proportional fitting. All population estimates and projections have a 30th June reference date.

Projections of the Australian population by birthplace, sex and single years of age were prepared in single year intervals from 2016 to 2056 using a birthplace-specific cohort-component population projection model created specifically for this project. The core of the projection model consists of the demographic accounting equation:

$$ {P}_{s,a+1}^i\left(t+1\right)={P}_{s,a}^i(t)-{D}_{s,a\to a+1}^i+{I}_{s,a\to a+1}^i-{E}_{s,a\to a+1}^i $$

in which:

*P* = population

*D* = deaths

*I* = immigration

*E* = emigration

*i* = birthplace

*s* = sex

*a* = age

*t* = point in time

*a*→*a*+1 = the period-cohort which ages from *a* to *a*+1 during the year.

Emigration and deaths are calculated as the product of rates and person-years at risk, for example:

$$ {D}_{s,a\to a+1}^i={d}_{s,a\to a+1}^i\ \frac{1}{2}\left[{P}_{s,a}^i(t)+{P}_{s,a}^i\left(t+1\right)\right] $$

in which:

*d* = death rate.

Because immigration is influenced more by migration policies than a population-at-risk, it is projected directly as flows. Births are projected in the usual way for a cohort-component model as the product of age-specific fertility rates and person-years at risk. The one difference is that all births from overseas-born women which occur in Australia are allocated to the Australia-born population.

$$ {B}^{Aus}=\sum \limits_i\sum \limits_a\left({b}_a^i\ \frac{1}{2}\left[{P}_{f,a}^i(t)+{P}_{f,a}^i\left(t+1\right)\right]\right) $$

in which:

*B* = births

*Aus* = Australian-born

*b* = fertility rate

*f* = females.

We produced projections for many individual countries and world regions of birth, but in order to present a clear picture of broad-level trends, we report projections for two groupings of countries based on the ABS classification of birthplaces (Australian Bureau of Statistics, 2016). The first is a broad 10 category grouping of birthplaces by continent or major sub-continental region (referred to as broad birthplace categories); the second is an expanded 26 category list consisting of a few countries with large and well-established immigration flows to Australia together with other world regions (referred to as main birthplace categories). Table 4 in the Appendix lists the countries/territories in each of the two birthplace groupings.

Our projection is a ‘business as usual’ scenario that assumes recent demographic trends continue into the future. No adjustments were made for the demographic impacts of the COVID-19 pandemic because at the time of writing, these were not fully clear, and our main focus is on long-term outcomes. Average fertility rates by birthplace of women for the 2011-2016 periods were assumed to remain constant for the whole projection horizon. Mortality was assumed to continue long-run trends, with assumptions based on birthplace and sex-specific life expectancy at birth values. A national life table projection was created for the Australian population overall based on Ediev’s (2008) mortality forecasting method, with birthplace-specific life expectancy assumed to move in concert with this projection but at fixed lower or higher values according to 2011-2016 life expectancy differentials. Birthplace-specific age-sex-specific death rates corresponding to assumed life expectancies were calculated from the national life tables (Wilson, 2018).

Country of birth immigration and emigration flow data by sex and single years of age were obtained from the ABS for the 2011-2016 base period. Minor adjustments to the volumes and age patterns of migration were made to obtain population accounting consistency—i.e. ensuring that the 2011 ERPs by birthplace, sex and age when subject to births, deaths, immigration and emigration over the 2011-2016 period resulted in (or were very close to) 2016 ERPs. In the projections, annual age-sex-specific immigration numbers were constrained to a national total immigration flow which continued the historical long-run increase in immigration over time. Base period emigration rates were assumed to remain constant. Implicit in these international migration assumptions are no major changes to migration policy settings or the broader economic and political environment.

The contributions of increasing life expectancy, international migration, and cohort flow to future population change were determined by running variant projections. Cohort flow refers to the effect of varying sizes of cohorts moving into the 65+ age group over time (incorporating the influence of mortality and international migration, plus births for the Australia-born only, shaping cohort sizes up until the jump-off year of 2016). The variants used exactly the same assumptions as the business as usual scenario with the exception of (i) fixed life expectancy assumptions in one variant, and (ii) no international migration in the second. The difference between the main projection and the variant excluding life expectancy change indicates the effect of life expectancy improvements, whilst the difference between the main projection and the no migration variant indicates the effect of international migration. The remaining growth we attribute to cohort flow. There is an approximation in this simple decomposition because we ignore the interaction of migration and mortality, but the overall picture it paints is reliable^1^.

Ethics approval for this project was provided by the Melbourne School of Population and Global Health (MSPGH) Human Ethics Advisory Group (Ethics ID: 2056200.1).

## Results

### The changing composition of Australia’s 65+ population

Over the 1996-2016 period, Australia’s population aged 65+ grew considerably, increasing from 2.19 million to 3.67 million, or by 68% (Australian Bureau of Statistics, 2019b). By way of comparison, the total resident population increased by 33% during this time. The Australia-born component of the 65+ population increased from 1.51 million to 2.29 million (+51%) whilst the overseas-born grew at a faster rate, increasing from 0.68 million to 1.38 million (+104%). Thus, the overseas-born share of the total 65+ population increased, growing from 30.9% in 1996 to 37.6% in 2016.

**Fig. 1:**
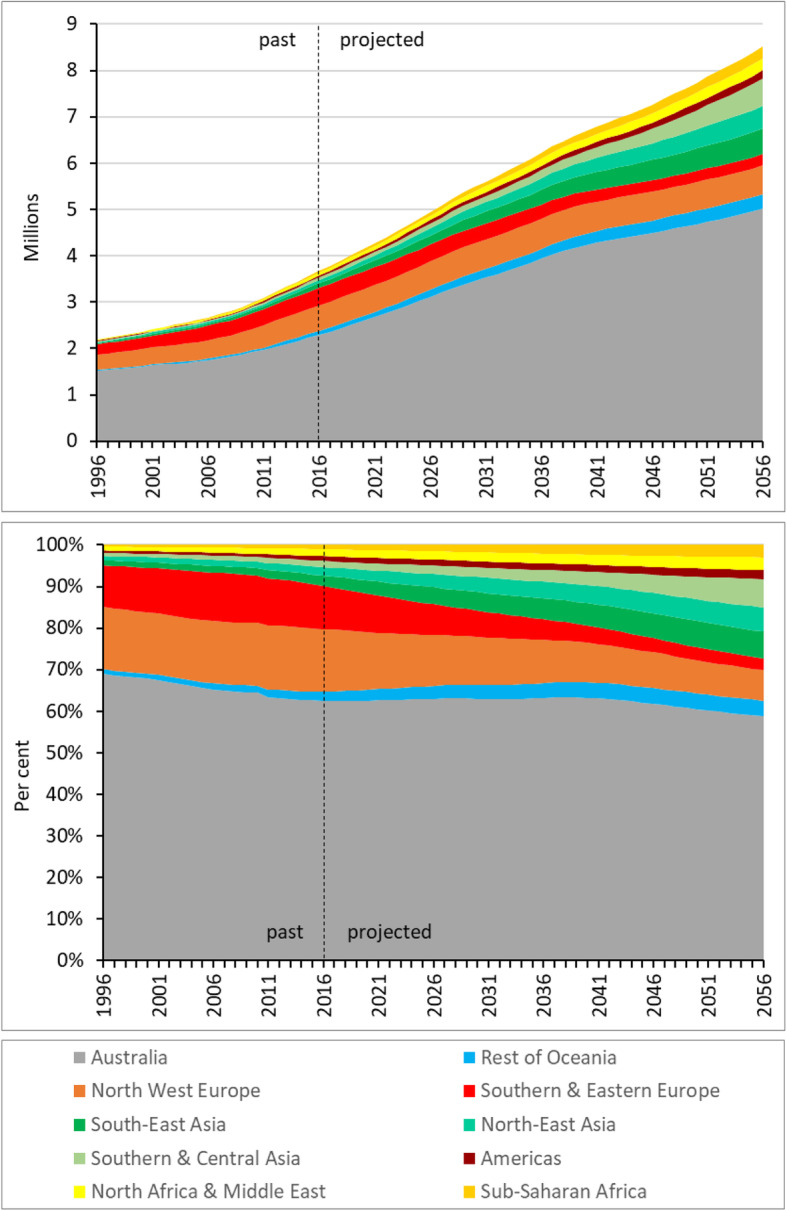
The past and projected population of Australia aged 65+ by broad birthplace category, 1996-2056. Sources: ABS; authors’ projections

Figure 1 illustrates the birthplace composition of Australia’s 65+ population by broad birthplace category from 1996 to 2056 using published ABS data from 1996 to 2016, and then our projections out to 2056. The top graph shows the absolute size of the birthplace populations whilst the lower graph contains the same information but expressed as a percentage of the 65+ population. Over the 1996-2016 period, all broad birthplace populations increased in size, with the North West Europe and Southern & Eastern Europe-born populations increasing by about the same percentage as the 65+ population overall, whilst all others increased by proportionally greater amounts. The result was a smaller proportion of the 65+ population born in Australia by 2016, little change in the proportions born in Europe, and increases for all other broad birthplace categories (lower graph in Fig. 1).

In the future, the 65+ population is expected to grow rapidly, reaching 8.5 million by 2056, indicating considerable numerical population ageing. As a proportion of the total population, this age group is projected to grow from 15.2% in 2016 to 20.9% by 2056, signifying moderate structural ageing. Most broad birthplace populations will grow in size, with the exception of those born in Southern and Eastern Europe, which is probably peaking around now (2020). Its 2016 size of 380,000 is projected to have dropped to 239,000 by 2056. The population born in North West Europe is projected to grow for the first half of the projection horizon, but then experiences a mixture of decline and growth in the second half. Over the 2016-2036 period, the highest growth rates are projected for the Asia-born and Sub-Saharan Africa-born 65+ populations (all over 200% growth). The South East Asia-born population grows from 95,000 in 2016 to 312,000 by 2036; equivalent figures are 75,000 and 253,000 for North East Asia, and 53,000 and 173,000 for Southern and Central Asia. In the later 2036-2056 period the Southern and Central Asia-born is expected to grow the most, increasing to 579,000 by 2056.

The differential growth of these populations will have a marked impact on the birthplace composition of the 65+ population, as the lower graph in Fig. 1 illustrates. The Europe-born and Australia-born populations will comprise a smaller share of the total 65+ population by 2056, whilst all other broad birthplace groups will have increased their share. By 2056, the Australia-born is projected to comprise 58.8% of the 65+ population (down from 62.4% in 2016), the Europe-born groups are collectively 10.1% (25.5% in 2016), whilst the Asia-born groups together contribute 19.1% (up from 6.0% in 2016).

Greater geographical detail is shown in Table 2, which summarises population estimates and projections for the 26 main birthplace categories. The 65+ population born in Southern Asia is projected to experience the greatest numerical increase over the projection horizon, increasing from 50,000 in 2016 to 520,000 by 2056 (+470,000). The Chinese Asia-born population is next (65,000 in 2016 to 430,000 by 2056), followed by Maritime South East Asia (56,000 to 301,000) and Mainland South East Asia (38,000 to 251,000). Proportionally, the largest projected increases are for two birthplace regions that start from a low base: Central and West Africa, which increases from 1000 to 29,000 during the course of the projections (+3783%), and Central Asia (3000 to 59,000 or + 2245%).

**Table 2:** The past and projected population of Australia aged 65+ by main birthplace category, 1996-2056

	Estimated		Projected		% change		
Country/region of birth	1996	2016	2036	2056	1996-2016	2016-2036	2036-2056
Australia	1,514,300	2,289,900	3,932,800	5,010,900	51	72	27
New Zealand	19,000	65,500	152,000	200,100	245	132	32
Melanesia	1200	3700	17,000	27,800	220	354	64
Micronesia	100	100	500	1000	67	449	81
Polynesia	2800	14,300	52,200	87,600	420	264	68
UK	252,200	402,700	527,200	509,700	60	31	−3
Ireland	10,500	17,100	20,800	34,700	63	22	67
Western Europe	63,800	125,100	87,700	67,900	96	−30	−23
Northern Europe	4000	9600	11,100	11,800	139	16	6
Southern Europe	91,300	165,300	112,100	71,300	81	−32	−36
South Eastern Europe	58,500	160,700	143,100	93,700	175	−11	−35
Eastern Europe	69,900	54,200	50,800	74,300	−22	−6	46
North Africa	9200	18,100	27,300	42,300	97	51	55
Middle East	11,500	44,200	119,800	217,000	284	171	81
Mainland South East Asia	11,600	38,200	147,400	251,300	229	285	71
Maritime South East Asia	13,100	56,400	164,800	301,500	330	192	83
Chinese Asia	20,300	65,400	223,400	429,600	222	241	92
Japan & the Koreas	2300	9100	29,600	65,700	289	225	122
Southern Asia	16,200	50,400	156,400	520,400	210	210	233
Central Asia	400	2500	16,700	58,900	597	565	253
North America	6,500	18,900	41,500	61,300	191	119	48
South America	3700	19,800	45,700	99,000	437	131	117
Central America	500	2100	8900	18,500	340	319	109
Caribbean	400	1300	3100	4700	215	149	50
Central and West Africa	100	700	8200	28,700	957	1002	252
Southern and East Africa	9100	36,800	121,000	226,300	305	229	87
Overseas born	678,100	1,382,300	2,288,200	3,505,200	104	66	53
Total	2,192,400	3,672,300	6,221,100	8,516,100	68	69	37

Sources: ABS; authors’ projections

Note: All population numbers have been rounded to nearest 100

The largest numerical decline over the projection horizon is expected to occur for the Southern Europe-born 65+ population which declines in number from 165,000 in 2016 to 71,000 in 2056, followed by the South Eastern Europe-born (161,000 to 94,000). Decline is also expected for the Western Europe-born. But some European-born older populations are projected to experience growth. The 65+ UK-born population increases for the first half of the projection horizon before declining slightly by 2056, whilst the Ireland-born population is expected to grow strongly. Another population with a long-established connection to Australia, the New Zealand-born, is also projected to grow strongly in the 65+ ages, growing from 66,000 to 200,000 by 2056.

**Fig. 2:**
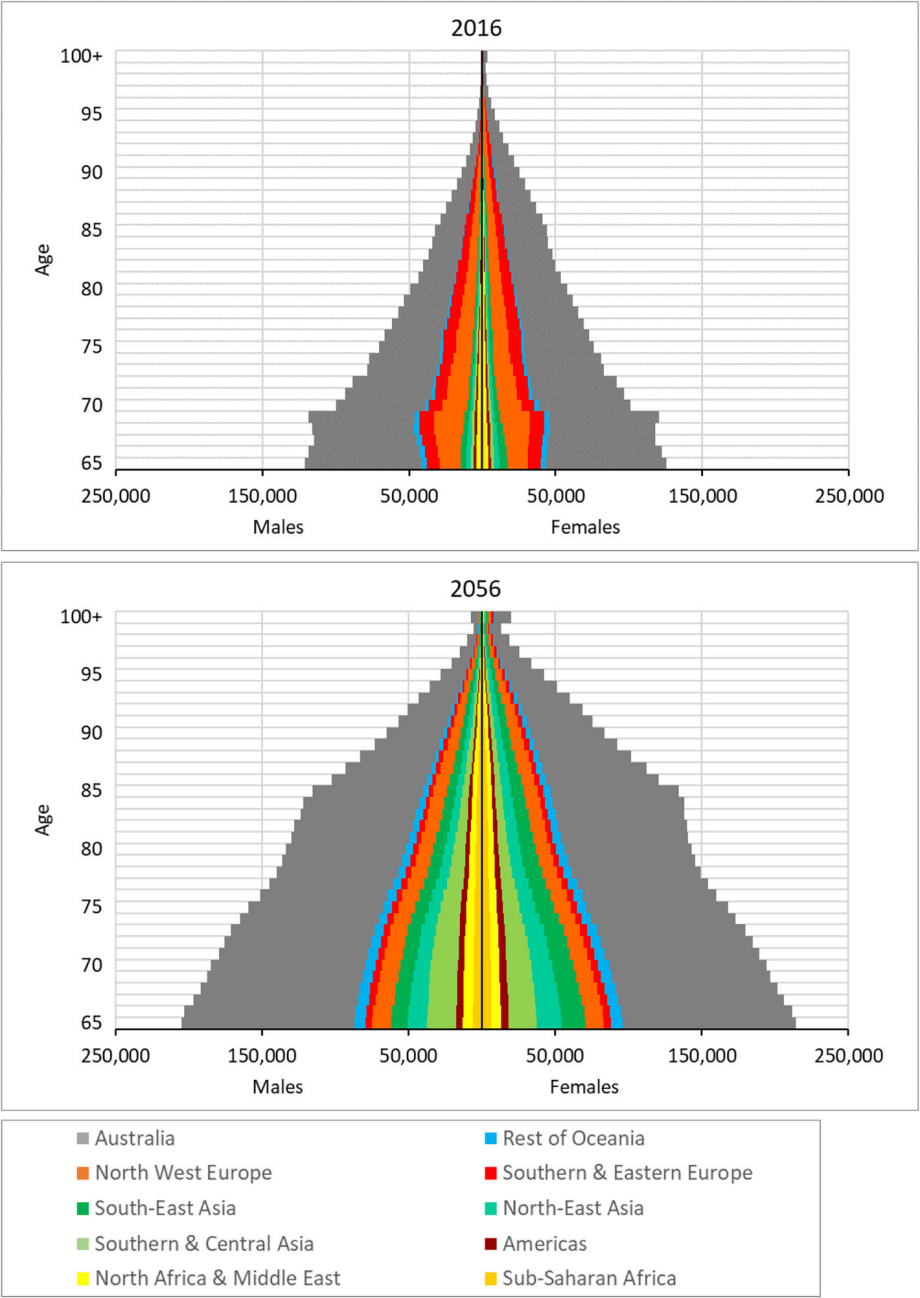
The age-sex structure of the 2016 (estimated) and 2056 (projected) older population of Australia by broad birthplace category. Sources: ABS; authors’ projections

The recent and projected age-sex structure of Australia’s older population by broad birthplace category is illustrated by the population pyramids in Fig. 2. In 2016 (upper graph), the overseas-born populations, shown by the non-grey colours, represent a large minority of the older population of Australia, with the European-born populations (shown in the orange and red) clearly standing out. The pyramidal shape is interrupted by the larger populations at ages 68 and 69 amongst the Australia-born and European-born. These populations represent the start of the baby boom born just after the end of World War 2. For most broad birthplace populations, there are more females than males in the 65+ age group, which to a large extent reflects differential mortality by sex. The North Africa and Middle East-born population is the one exception as a consequence of male-dominated immigration flows. For the overseas-born as a whole there are 93 males per 100 females in 2016 in the 65+ age group.

By 2056, the older population is projected to be much larger and more diverse (lower graph). The European-born populations clearly represent a smaller proportion, whilst those born in Asia, Africa, and the Rest of Oceania are much more prominent. The slightly bigger Australia-born age groups around age 85 are due to large birth cohorts born in the early 1970s. By 2056, the sex ratio of the Australia-born older population is projected to have increased, reflecting assumed convergence of male and female mortality rates over time. The overseas-born population overall is projected to have a sex ratio of 89 males per 100 females amongst the 65+ population by 2056, slightly lower than in 2016. For individual broad birthplace populations, there is a mix of increases and decreases in sex ratios into the future. The causes lie in sex imbalances in international migration flows.

### Drivers of future demographic change

The proximate demographic drivers of projected growth in the 65+ birthplace populations consist of increasing life expectancy, international migration, and cohort flow. The projection of the 65+ population for all birthplaces gives a total of 8.52 million by 2056, an increase of 4.84 million since 2016. Excluding mortality change results in a total of 7.11 million, and no international migration gives 7.92 million. Therefore, the effect of improving life expectancy on the growth of the 65+ age group between 2016 and 2056 is 1.40 million (29% of growth), whilst the impact of international migration is 0.59 million (12%). The remainder, 59% of projected growth, is due to cohort flow into the 65+ age group from younger ages over the course of the projection horizon.

**Fig. 3:**
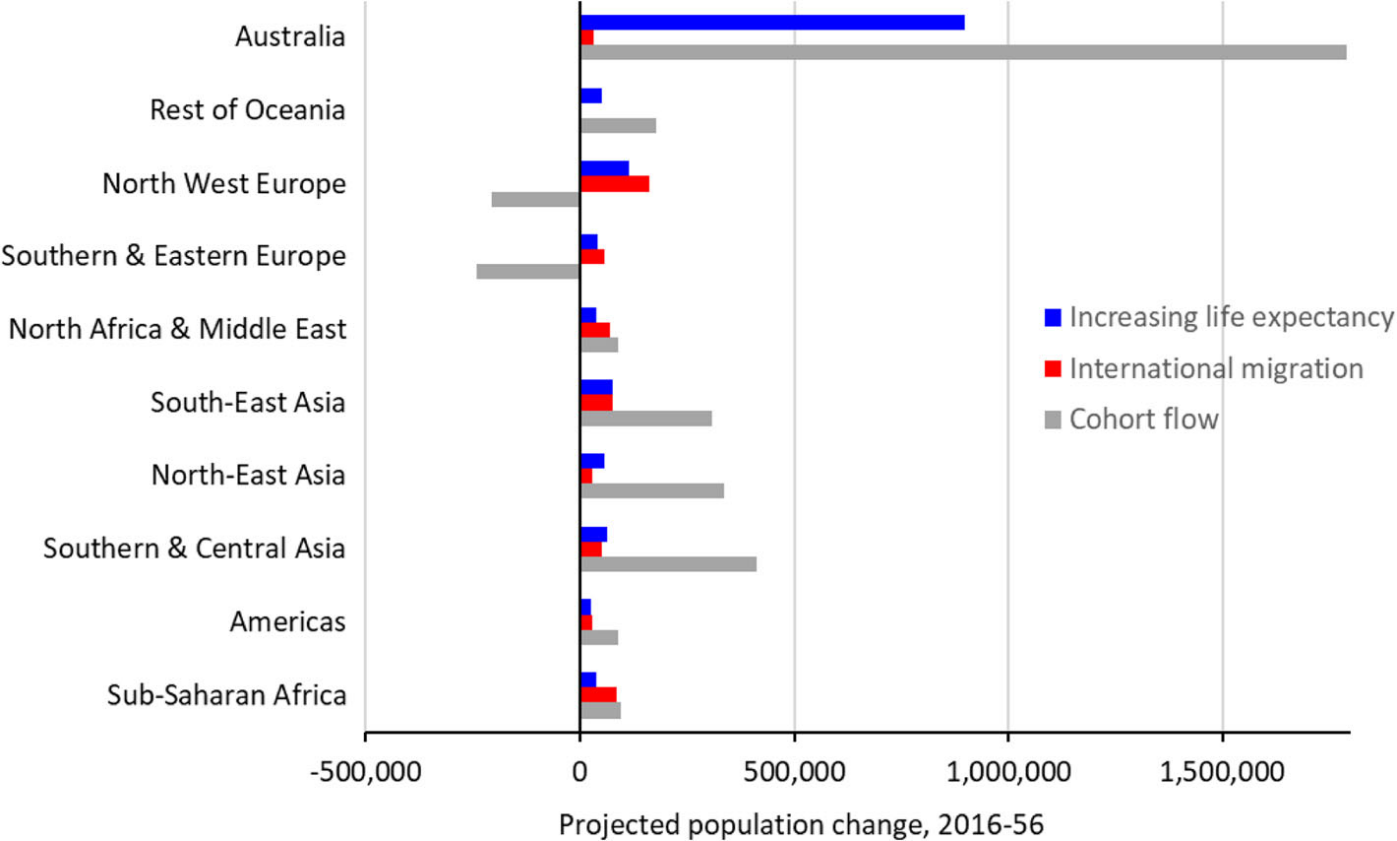
The demographic drivers of population change at ages 65+ by broad birthplace category, 2016-2056. Source: authors’ projections

Figure 3 presents the same decomposition by broad birthplace category. It shows the contributions to projected growth from 2016 to 2056 of the 65+ population from increasing life expectancy, international migration, and cohort flow from younger ages. For all broad birthplace categories, the contribution of international migration is positive, though mostly modest. As would be expected, increases in life expectancy also contribute to population growth in all birthplace categories over time. However, the most important factor, and the one most responsible for the differential growth of the birthplace populations, is cohort flow. For the Australia-born, this is substantial, as would be expected for a population that is growing rapidly. In particular, during the first two decades of the projection horizon, large baby boom cohorts are reaching their 65th birthdays. For the Asia-born populations, cohort flow is by far the most important contributor to projected growth. These populations had relatively small numbers in the older age groups in 2016, but the substantial immigration of young adults in recent years means the size of cohorts reaching age 65 during the 2016 to 2056 period increases rapidly. In contrast, for those born in North West Europe and Southern and Eastern Europe, the cohort flow effect is negative. In other words, cohorts from these birthplaces reaching age 65 will be smaller and smaller over the course of the projection horizon.

More geographical detail is presented in Table 3, which quantifies the projected drivers of population growth at older ages for the 26 main birthplace categories. As the table shows, there is a little more variation in the international migration contributions. For a few birthplace groups—those born in Ireland, New Zealand, Western Europe, Northern Europe, Japan and the Koreas, and North America—migration makes a negative contribution to growth. This means that, within the 65+ age group and at younger ages which have cohorts that reach age 65 during the projection horizon, there is net international migration loss overall. As a proportion of growth, the effect of migration is greatest for the Ireland-born, and at least part of this is likely to be due to return migration (Barrett, 2002; O’Leary & Negra, 2016). Cohort flow is only negative for some European-born birthplace groups. It is the dominant driver of growth for the New Zealand-born and most Asia-born populations and is numerically greatest for those born in Southern Asia and Chinese Asia. For the Middle East-born and Southern and East Africa-born, all three drivers of growth make substantial contributions to overall growth.

**Table 3:** The demographic drivers of population change for ages 65+ by main birthplace category, 2016-2056

	Increasing	International	Cohort flow	Total
Country/region of birth	Life expectancy	Migration		
Australia	898,800	32,400	1,789,800	2,721,000
New Zealand	32,200	−35,700	138,100	134,600
Melanesia	4800	9800	9500	24,100
Micronesia	200	300	400	900
Polynesia	13,400	29,000	30,900	73,300
UK	95,600	174,000	−162,500	107,100
Ireland	4900	−10,400	23,200	17,600
Western Europe	11,500	−2400	−66,400	−57,300
Northern Europe	2000	−700	1000	2200
Southern Europe	12,900	26,300	−133,200	−94,000
South Eastern Europe	18,400	10,000	−95,400	−67,000
Eastern Europe	10,800	20,100	−10,800	20,100
North Africa	6500	12,500	5300	24,300
Middle East	32,800	57,300	82,600	172,800
Mainland South East Asia	34,900	35,600	142,600	213,100
Maritime South East Asia	41,900	39,100	164,100	245,100
Chinese Asia	47,200	52,000	264,900	364,100
Japan and the Koreas	8700	−23,000	71,000	56,600
Southern Asia	55,400	23,900	390,700	470,000
Central Asia	7800	27,900	20,700	56,400
North America	7900	−8600	43,100	42,300
South America	13,700	26,100	39,400	79,300
Central America	2700	7400	6200	16,400
Caribbean	800	2300	400	3400
Central and West Africa	3800	11,500	12,600	28,000
Southern and East Africa	33,500	74,800	81,200	189,500
Overseas born	504,100	559,300	1,059,500	2,122,900
Total	1,402,900	591,700	2,849,300	4,843,900

Source: authors’ projections

Note: All numbers have been rounded to nearest 100

## Discussion

These projections indicate substantial future growth in the size of the older population of Australia, along with a major shift in the birthplace origins of this population. Many of the overseas-born are from non-English-speaking countries. Therefore, to achieve equity in service provision, people from CALD backgrounds must be included in the planning, provision, and regulation of aged care services. This imperative is expressed in policy documents such as the Australian Department of Health’s Aged Care Diversity Framework, which states that it is a right of all Australians, regardless of background or life experience, to be able to access good, inclusive care (Department of Health, 2017). Further, the Department’s guide for age care providers around the care of older CALD people stresses the importance that this care be culturally appropriate (Department of Health, 2019 ).

However, what is culturally appropriate depends on CALD communities’ culturally derived beliefs and attitudes, and health and digital literacy levels, including an understanding of how health and care systems work (Shanley et al., 2012). Whilst European migrants currently make up a large portion of the CALD Australians aged over 65, this will change over the coming years with the Asia-born and Africa-born being the fastest growing CALD populations (Fig. 1; Table 2). Whilst ethnic-specific aged care has an important role to play, with an increasingly multicultural and ageing population, it is unwise to solely rely on ethnic-specific services to provide aged care to CALD Australians. Rather, both ethnic-specific and mainstream care models must be inclusive of all the Australian communities they serve.

In addition, the challenge facing ethnic-specific services is that, contingent on demand, some will be obsolete whilst others will need to develop and scale up. This is an issue of equity and access as well as business continuity. Further research is needed into how the needs of CALD communities which currently lack access to ethnic-specific care can be effectively serviced by mainstream aged care providers, how ethnic-specific services that will be redundant can transition to caring for other communities or be future-proofed in other ways, and how sustainable, culturally appropriate models of care can be developed and delivered in the long-term.

The projections described above also have major implications for addressing language barriers, increasing awareness of aged-related diseases, such as dementia, and care options in CALD communities. They are also highly relevant to the creation of a skilled multicultural aged care workforce. A crucial issue will be growing the aged care workforce, which must triple its workforce to almost 1 million by 2050 because of population ageing (Department of Jobs and Small Business, 2018). Migrant care workers are already strongly represented in aged care; for example, a 2016 survey showed 40% of recent employees in residential aged care facilities were migrants (Commonwealth of Australia, 2017), primarily working as personal care attendants (PCAs). Most PCAs are from Indian and Filipino backgrounds, but there is increasing representation from new and emerging communities such as those from Iraq and Sudan (Commonwealth of Australia, 2017; Nichols, Horner, & Fyfe, 2015). Thus, concomitant with an increasingly culturally diverse older population is an increasingly multicultural aged care workforce, which may or may not be matched to the groups it must serve (Adebayo, Nichols, Heslop, & Brijnath, 2019). Responding to these challenges necessitates interventions at community, service-provider and systems levels to understand existing inadequacies. Some solutions may be possible via community collaboration and codesign. Others, such as culturally appropriate services, interpreters with relevant expertise, and culturally adapted materials, need to be addressed on the side of policy and services (Federation of Ethnic Communities’ Councils of Australia, 2016).

At this point, we note that birthplace composition in the future may not prove to be a reliable proxy on the extent to which certain share of older migrants cannot speak English. For example, with globalisation and the current speed of social transformation unfolding across the globe, it is likely to be the case that more and more potential migrants from non-English speaking countries will be more proficient in English than previous generation of migrants. With the ongoing selective migration programme that favoured skilled migrants, the ability to communicate effectively in English is not likely to be a concern for a growing proportion of older migrants in Australia. But, importantly, governments and service providers should not falsely assume that if it only allows those with high English proficiency to migrate then language barriers will be ameliorated in the end. Ageing and age-related conditions such as dementia can degrade people’s ability to communicate in their non-primary languages (Nickels, Hameau, Nair, Barr, & Biedermann, 2019; Tipping & Whiteside, 2015). Thus, whilst there may be high levels of English proficiency in some CALD communities, language barriers may still be present for the CALD person with dementia in aged care. Communication difficulties can amplify social isolation, depression, and unmet need (Runci, Eppingstall, van der Ploeg, & O’Connor, 2014), which can leave an older CALD person with dementia vulnerable to an increased risk of experiencing abuse.

Thus, the future aged care workforce, which itself is increasingly multicultural needs strong communication skills. The workforce also needs to be culturally competent, i.e. able to deliver culturally appropriate and accessible care (Mollah, Antoniades, Lafeer, & Brijnath, 2018; Thackrah & Thompson, 2013). Technology has a role to play but the person-centred nature of care work means that technology cannot be a panacea for human contact. As migrant care workers grow as a proportion of the aged care workforce, leveraging a multilingual, multicultural workforce can help build connections between vulnerable and diverse groups. With its diverse, multicultural population, Australia has a unique opportunity to develop innovative models for caring for diverse older people; such models will be relevant to the ageing populations of many high-income countries. Indeed, prior Australian studies have noted the importance of acknowledging the heterogenous nature of needs for aged care, health, and support services for older Australians from culturally and linguistically diverse backgrounds (Haralambous et al., 2014; Henderson & Kendall, 2011; Kanitsaki, 2003; Kourbelis, 2016; Radermacher et al., 2009; Rao et al., 2006; Williams & Harris, 2010). Echoing these studies, the projected increases in both the size and diversity of older Australians, our findings underline the significant policy, funding, and planning challenges ahead.

The projected changes to the older migrant populations of Australia requires gerontologists, demographers, and other researchers in ageing to take these changes seriously. Despite CALD Australians making up over a third of the older population, they are often excluded from Australian research, and information on ethnicity, language, and country of birth is not routinely collected or reported (Low, Barcenilla-Wong, & Brijnath, 2019). For example, data from 16 Australian dementia epidemiology studies were collected only in English and six of these studies excluded participants not fluent in English (Low, Barcenilla-Wong, & Brijnath, 2019). This scenario also manifests in other countries such as the USA, the UK, and Canada. Potential consequences of this under-representation are non-representative samples in epidemiology and clinical trial studies resulting in inaccurate effect sizes on which health, policy and economic decisions are based, and less effective implementation of research evidence into practice for CALD communities. The task for researchers is to develop appropriate measures to define ‘CALD’, ones that sufficiently account for heterogeneity and that are integrated across all government funded surveys such as those administered by the Australian Bureau of Statistics and the Australian Institute of Health and Welfare.

At a community level, it remains to be seen whether the growing diversity of Australia’s older population would be accompanied by a growing differentiation between the social and economic capacity of migrant communities and families to support their older members. For example, previous studies have highlighted significant variations in the rate of language maintenance amongst later generation migrants in Australia (Karidakis & Arunachalam, 2016; Khoo, 2003). With documented language reversion amongst older people from culturally and linguistically diverse communities (Haralambous et al., 2014; Tipping & Whiteside, 2015), there is a need for policy support to promote the maintenance of languages other than English amongst families and communities in an increasingly diverse Australia.

Overall, the implications of population ageing and diversity in Australia are similar to those currently facing other major destination countries such as the UK, France, Germany, the USA, Canada, and New Zealand. On the one hand, increasing diversity in the older population may present significant challenges attributed to barriers to care and support in later life as outlined above. On the other hand, it may present novel well-being enhancing opportunities in the forms of wider transnational networks and lifestyle options amongst older Australians. Earlier studies had identified the emergence of economically and socially beneficial transnational family and kinship networks from the migration of highly skilled migrants to Australia and the New Zealand (Baldassar, 2017; Brijnath, 2009, 2020; Gilbert, Antoniades, & Brijnath, 2019; Ho, 2002; Parr, Lucas, & Mok, 2000). The advent and proliferation of digital communication technology may also serve to maintain distant support networks and amplify diverse local connections amongst elderly migrants in Australia (Baldassar & Wilding, 2019; Millard, Baldassar, & Wilding, 2018).

## Conclusions

This paper has examined recent past changes in Australia’s older population by birthplace group and presented new projections from 2016 to 2056. Our key findings include (i) fast growth of the 65+ population occurred between 1996 and 2016, with the overseas-born share increasing; (ii) continued rapid growth is likely over coming decades; (iii) the birthplace composition of the overseas-born will undergo a major shift from being European-dominated now to being Asian-dominated by mid-century and (iv) the key demographic factor affecting the size of birthplace-specific older populations will be cohort flow. We have also discussed many of the policy implications of these coming demographic changes for aged care, health care, language services, the aged care workforce, regulatory frameworks and research needs in demography and gerontology.

Our study contains a number of limitations, of course. Whilst the results of a ‘business as usual’ projection have been presented, reality will inevitably deviate from that scenario to some extent. The impact of COVID-19 and the short-term closure of many international borders is one major example, and we have not made allowance for that in our projections. The short-run effects on international migration will be a large drop in the number of migrants, but the longer-term impacts are very difficult to determine at the time of writing. It appears, at present, that the impact of the pandemic on mortality in Australia will be small. However, as Fig. 3 demonstrates, the major factor affecting the size of future 65+ birthplace populations in Australia is cohort flow. In other words, the size of these older populations will be largely influenced by the size of younger migrant cohorts—already resident in Australia—moving up into the 65+ age group over time. Net international migration is relatively low above age 45, so for at least the first 20 years of the projection horizon, migration has little impact on the projections. In addition, mortality is relatively predictable. As a consequence, we can be fairly confident about the accuracy of the projections of older birthplace population sizes for this period. Further into the future, the projections are increasingly less reliable. Projections of the *proportion* of populations aged 65+, however, are less reliable throughout the projection horizon. This is because there is far more uncertainty surrounding the future growth of overseas-born childhood and younger adult populations due to uncertainty about the future trajectory of international migration.

Future demographic work on ageing and diversity in Australia should explore the role of age at migration in driving variations in later life well-being outcomes, comparing the experience of older migrants who came during their young adulthood or earlier compared to those who came at older ages through family reunion schemes. In addition, considerations concerning the future challenges around ageing-related health and service provisions should better account for future patterns of intermarriage between overseas-born and Australia-born populations (see Khoo, 2004, 2011; Khoo, Birrell, & Heard, 2009). More broadly, further work is needed to cross examine the potential implications of our findings on the body of work around the subnational aspects of ageing (Hugo, 2008; Winterton & Warburton, 2012), the provision of care and services for older migrant populations (Clarke & Isphording, 2017; Furnham & Shiekh, 1993; Rao et al., 2006; Tipping & Whiteside, 2015) and transnational care-giving practices in Australia (i.e. migrant families’ ability to care for their elderly members across national borders see Baldassar, 2007; Merla, 2015). From a demographic modelling perspective, future research needs include endogenizing second generation migrants into our projections model and incorporating suggested improvements in international migration forecasting in Australia (Temple & McDonald, 2018; Wilson, 2017). Extensions to subnational regions and smaller areas could also be considered given that the planning for many services is regional and local in scale. Finally, to align more closely with planning for service provision, the projections could also be extended to include a breakdown by English language proficiency.

## Data Availability

The population projections presented in the paper are available from figshare at 10.6084/m9.figshare.12720920.v1.

## Appendix

**Table 4:** The classification of countries of birth

Broad category	Country/region	Constituent countries/territories
Australia	Australia	Australia
Rest of Oceania	New Zealand	New Zealand
	Melanesia	New Caledonia, Papua New Guinea, Solomon Islands, Vanuatu
	Micronesia	Guam, Kiribati, Marshall Islands, Federated States of Micronesia, Nauru, Northern Mariana Islands, Palau
	Polynesia	Cook Islands, Fiji, French Polynesia, Niue, Samoa, American Samoa, Tokelau, Tonga, Tuvalu, Wallis and Futuna, Pitcairn Islands
North West Europe	UK	UK, Channel Islands, Isle of Man
	Ireland	Ireland
	Western Europe	Austria, Belgium, France, Germany, Liechtenstein, Luxembourg, Monaco, Netherlands, Switzerland
	Northern Europe	Denmark, Faroe Islands, Finland, Greenland, Iceland, Norway, Sweden, Aland Islands
Southern and Eastern Europe	Southern Europe	Andorra, Gibraltar, Holy See, Italy, Malta, Portugal, San Marino, Spain
	South Eastern Europe	Albania, Bosnia and Herzegovina, Bulgaria, Croatia, Cyprus, North Macedonia, Greece, Moldova, Romania, Slovenia, Montenegro, Serbia, Kosovo
	Eastern Europe	Belarus, Czech Republic, Estonia, Hungary, Latvia, Lithuania, Poland, Russian Federation, Slovakia, Ukraine
North Africa and Middle East	North Africa	Algeria, Egypt, Libya, Morocco, Sudan, Tunisia, Western Sahara, Spanish North Africa, South Sudan
	Middle East	Bahrain, Gaza Strip and West Bank, Iran, Iraq, Israel, Jordan, Kuwait, Lebanon, Oman, Qatar, Saudi Arabia, Syria, Turkey, United Arab Emirates, Yemen
South East Asia	Mainland South East Asia	Myanmar, Cambodia, Laos, Thailand, Vietnam
	Maritime South East Asia	Brunei Darussalam, Indonesia, Malaysia, Philippines, Singapore, Timor-Leste
North East Asia	Chinese Asia	China (excludes SARs and Taiwan), Hong Kong (SAR of China), Macau (SAR of China), Mongolia, Taiwan
	Japan and the Koreas	Japan, Democratic People’s Republic of Korea (North), Republic of Korea (South)
Southern and Central Asia	Southern Asia	Bangladesh, Bhutan, India, Maldives, Nepal, Pakistan, Sri Lanka
	Central Asia	Afghanistan, Armenia, Azerbaijan, Georgia, Kazakhstan, Kyrgyzstan, Tajikistan, Turkmenistan, Uzbekistan
Americas	North America	Bermuda, Canada, St Pierre and Miquelon, United States of America
	South America	Argentina, Bolivia, Brazil, Chile, Colombia, Ecuador, Falkland Islands, French Guiana, Guyana, Paraguay, Peru, Suriname, Uruguay, Venezuela
	Central America	Belize, Costa Rica, El Salvador, Guatemala, Honduras, Mexico, Nicaragua, Panama
	Caribbean	Anguilla, Antigua and Barbuda, Aruba, Bahamas, Barbados, Cayman Islands, Cuba, Dominica, Dominican Republic, Grenada, Guadeloupe, Haiti, Jamaica, Martinique, Montserrat, Puerto Rico, St Kitts and Nevis, St Lucia, St Vincent and the Grenadines, Trinidad and Tobago, Turks and Caicos Islands, British Virgin Islands, United States Virgin Islands, St Barthelemy, St Martin (French part), Bonaire, Sint Eustatius and Saba, Curacao, Sint Maarten (Dutch part)
Sub-Saharan Africa	Central and West Africa	Benin, Burkina Faso, Cameroon, Cabo Verde, Central African Republic, Chad, Republic of Congo, Democratic Republic of Congo, Cote d’Ivoire, Equatorial Guinea, Gabon, Gambia, Ghana, Guinea, Guinea-Bissau, Liberia, Mali, Mauritania, Niger, Nigeria, Sao Tome and Principe, Senegal, Sierra Leone, Togo
	Southern and East Africa	Angola, Botswana, Burundi, Comoros, Djibouti, Eritrea, Ethiopia, Kenya, Lesotho, Madagascar, Malawi, Mauritius, Mayotte, Mozambique, Namibia, Reunion, Rwanda, St Helena, Seychelles, Somalia, South Africa, Swaziland, Tanzania, Uganda, Zambia, Zimbabwe

Source: https://www.abs.gov.au/ausstats/abs@.nsf/Lookup/2901.0Chapter1102016

## Abbreviations

ABS: Australian Bureau of Statistics
CALD: Culturally and linguistically diverse
ERPs: Estimated resident populations
PCAs: Personal care attendants

## Footnotes

1 We ran an additional projection with no migration and constant mortality and compared our residual cohort flow estimate with the growth from this projection. The numbers were very similar.
